# Polyunsaturated fatty acids: an alternative to statins for treating heart disease?

**Published:** 2013-11

Cardiovascular diseases (CVDs) are a group of common, debilitating conditions that can be fatal without treatment. Build-up of lipid in the arteries (atherosclerosis) is associated with increased risk of CVD, so lowering serum cholesterol is a major therapeutic route. This is achieved by administration of statins, which inhibit endogenous cholesterol synthesis. Although this therapy is effective, its use is limited in some cases because of side effects. Here, Amnon Schlegel and colleagues bring to light a possible alternative to statins for the treatment of CVD. Using a zebrafish model, they demonstrate that coenzyme-A-activated polyunsaturated fatty acids (PUFACoAs) can inhibit cholesterol synthesis. In support of this finding, they show that PUFA-CoAs can directly inhibit the human enzyme for cholesterol synthesis *in vitro*, and also have an inhibitory effect in mice. Thus, fish oil supplements that contain polyunsaturated fatty acids could represent a new therapeutic candidate for lowering blood cholesterol. This intriguing possibility now awaits confirmation in a clinical setting. **Page 1365**

**Figure:**
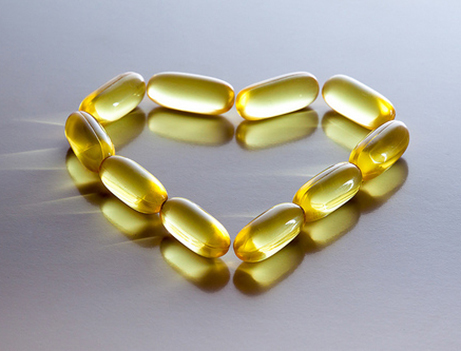
Photo: Creative Commons/Jo Christian Oterhals Flickr photostream

